# Associations Between Types of Balance Performance in Healthy Individuals Across the Lifespan: A Systematic Review and Meta-Analysis

**DOI:** 10.3389/fphys.2018.01366

**Published:** 2018-09-28

**Authors:** Rainer Kiss, Simon Schedler, Thomas Muehlbauer

**Affiliations:** ^1^Department of Health and Social Affairs, FHM Bielefeld-University of Applied Sciences, Bielefeld, Germany; ^2^Division of Movement and Training Sciences/Biomechanics of Sport, University of Duisburg-Essen, Essen, Germany

**Keywords:** postural control, children, adolescents, adults, correlation

## Abstract

**Background:** The objective of this systematic review and meta-analysis was to quantify and statistically compare correlations between types of balance performance in healthy individuals across the lifespan.

**Methods:** Literature search was performed in the electronic databases PubMed, Web of Science, and SPORTDiscus. Studies were included if they investigated healthy individuals aged ≥6 years and reported measures of static/dynamic steady-state, proactive, and/or reactive balance. The included studies were coded as follows: age group, gender, and balance type, test, parameter. Pearson's correlation coefficients were extracted, transformed (i.e., Fisher's z-transformed *r*_*z*_-value), aggregated (i.e., weighted mean *r*_*z*_-value), back-transformed to *r*-values, classified according to their magnitude, and statistically compared. The methodological quality of each study was assessed using the Appraisal tool for Cross-Sectional Studies.

**Results:** We detected twenty-six studies that examined associations between types of balance and exclusively found small-sized correlations, irrespective of the age group considered. More specifically, the weighted mean *r*_z_-values amounted to 0.61 (back-transformed *r*-value: 0.54) in old adults for the correlation of dynamic steady-state with proactive balance. For correlations between dynamic and static steady-state balance, the weighted mean *r*_z_-values amounted to 0.09 in children (*r*-value: 0.09) and to 0.32 in old adults (*r*-value: 0.31). Further, correlations of proactive with static steady-state balance revealed weighted mean *r*_z_-values of 0.24 (*r*-value: 0.24) in young adults and of 0.31 (*r*-value: 0.30) in old adults. Additionally, correlations between reactive and static steady-state balance yielded weighted mean *r*_z_-values of 0.21 (*r*-value: 0.21) in young adults and of 0.19 (*r*-value: 0.19) in old adults. Moreover, significantly different correlation coefficients (*z* = 8.28, *p* < 0.001) were only found for the association between dynamic and static steady-state balance in children (*r* = 0.09) compared to old adults (*r* = 0.31). Lastly, we detected trivial to considerable heterogeneity (i.e., 0% ≤ I^2^ ≤ 83%) between studies.

**Conclusions:** Our systematic review and meta-analysis showed exclusively small-sized correlations between types of balance performance across the lifespan. This indicates that balance performance seems to be task-specific rather than a “general ability.” Further, our results suggest that for assessment/training purposes a test battery/multiple exercises should be used that include static/dynamic steady-state, proactive, and reactive types of balance. Concerning the observed significant age differences, further research is needed to investigate whether they are truly existent or if they are caused by methodological inconsistencies.

## Introduction

In everyday life, adequate postural control is needed to safely manage activities of daily living (e.g., walking to school/college or climbing the stairs to one's office without sustaining a fall) and to regularly engage in sports-related activities (i.e., riding a bicycle or engaging in team sports). Thus, balance performance represents an important health- and activity-related component of everyday life that is also relevant across the human lifespan (Woollacott and Shumway-Cook, 1990; Granacher et al., 2011b). On the other hand, deficits in balance performance have been identified as important intrinsic factors increasing the risk of falling and sustaining an injury in children (Razmus et al., 2006), adolescents (Wang et al., 2006), young (Fousekis et al., 2011), and older adults (Rubenstein, 2006).

According to Shumway-Cook and Woollacott (2016) as well as, balance control involves static conditions in which the base of support (i.e., feet), and the ground remain stationary, as well as dynamic conditions in which both the base of support and the center of mass shift. Further, balance performance can further be subdivided into four types. These include, static steady-state balance (i.e., maintaining a steady position while sitting or standing), dynamic steady-state balance (i.e., maintaining a steady position while walking), proactive balance (i.e., anticipation of a predicted postural disturbance), and reactive balance (i.e., compensation of an unpredicted postural disturbance) (Shumway-Cook and Woollacott, 2016). This classification has widely been used in research on postural control in various populations (Bohannon, 2006; Springer et al., 2007), implying that these components represent different types of balance that are hardly associated and only show small-sized correlations among each other. On the other hand, balance performance has, particularly in textbooks (Fleishman, 1964; Schnabel et al., 2014; Meinel and Schnabel, 2018) used in college education, been introduced as a “general ability,” suggesting that the various types of balance are highly interlinked. That is, a person with good static steady-state balance (e.g., less postural sway during one-legged stance) is supposed to also show superior performance in a dynamic steady-state balance task (e.g., fast gait speed during figure-eight walk). This implies large-sized correlations among the above-mentioned four types of balance because they are representatives of one balancing ability.

Additionally, the process of aging seems to have an effect on the association between types of balance in healthy individuals. In general, it has been reported that, depending on the parameter investigated, postural control shows a U-shaped trend for static (i.e., postural sway) or an inverted U-shaped course for dynamic (i.e., gait speed) steady-state balance performance across the lifespan (Granacher et al., 2011b). These age-related changes in balance performance are particularly based on the underlying neurophysiological structures responsible for postural control (Woollacott and Shumway-Cook, 1990). In children, the neuromuscular system is still developing due to maturation of the central nervous system (e.g., sensory integration) and has not reached its full functionality (Shumway-Cook and Woollacott, 1985; Woollacott and Shumway-Cook, 1994). In old adults, the neuromuscular system is in a state of functional decline and has lost its full capability due to, for example, a decline in the number of motor neurons and a diminished sensory feedback (Bouche et al., 1993; Terao et al., 1996; Maisonobe et al., 1997). These maturation-/aging-related limitations in postural control may contribute to age differences in the correlation between types of balance performance. Depending on the maturation-/aging-related limitation, one specific type of balance performance might be more affected than other components. Proof for this notation comes from studies that applied the Sensory Organization Test (SOT) to different age groups (Hirabayashi and Iwasaki, 1995; Peterson et al., 2006; Steindl et al., 2006). For example, Peterson et al. (2006) investigated the use of specific sensory information in maintaining postural stability in healthy children (6–12 years) and in adults (20–22 years). They found less postural stability in children compared to adults in test conditions using visual (i.e., normal vision, support sway-referenced) and vestibular (i.e., eyes closed, support surface sway-referenced) information. However, no age differences were detected in the condition using somatosensory information (i.e., eyes closed, fixed support). These results might indicate that age-related differences in the associations between balance dimension exist since adults performed equally-well in each test condition while children aged 6–12 years showed diverging performances.

Thus, the aim of this systematic literature review and meta-analysis was to quantify and statistically compare associations between types of balance performance in healthy individuals across the lifespan. The classification of balance performance in various types implies that balance is task-specific and thus small-sized correlations among types of balance are expected. Contrary, the use of balance performance in terms of a “general ability” suggests large-sized correlations among types of balance. Additionally, we assume age differences for the association between types of balance performance.

## Methods

### Search of literature

We performed a computerized systematic literature search in PubMed, Web of Science, and SPORTDiscus up to May 2018. The following Boolean search strategy was applied using the operators AND, OR, NOT: {[postural balance (MeSH) OR posture (MeSH)] AND (correlation study OR association OR relationship) NOT (patients OR disease)]}. With respect to the PubMed database, Medical Subject Headings (MeSH) were used as it was indicated before. The search was limited to: English language, human species, and to full text original articles. Further, we analyzed relevant review articles (Hrysomallis, 2007; Zemkova, 2014; Muehlbauer et al., 2015) in an effort to identify additional suitable studies for inclusion in the database.

### Criteria for selection

Studies were considered eligible to be included if they met the following criteria: (a) participants had to be healthy subjects, (b) participants were aged ≥6 years, and (c) outcomes from at least two types of balance had to be tested in the study. Studies were excluded if: (a) they investigated patients or people with diseases, (b) it was not possible to extract correlation coefficients from the results section or (c) authors did not reply to our inquiries sent by email. Based on the predefined inclusion and exclusion criteria, two independent reviewers (SS, TM) screened potentially relevant articles by analysing titles, abstracts, and full texts to determine their eligibility. If SS and TM did not reach a consensus concerning inclusion of a study, a third reviewer (RK) was contacted for clarification.

### Study coding

Each study was coded for the following variables: number of participants, sex, and chronological age. Further, we coded type, test, and parameter for the assessment of balance performance. With respect to the classification of postural control published by Shumway-Cook and Woollacott (2016), balance performance was separated into four types: static steady-state (i.e., maintenance of a steady position while standing), dynamic steady-state (i.e., maintenance of a steady position while walking), proactive (i.e., anticipating an expected postural disturbance), and reactive balance (i.e., compensating an unexpected postural disturbance). If several parameters were reported within one type of balance, the most representative measure was used for further analysis. In terms of dynamic steady-state balance, gait speed was used. With regards to static steady-state balance, center of pressure (CoP) displacement during one-legged stance was defined as the most relevant parameter. Concerning proactive balance, maximal reach distance in the Functional-Reach-Test was used. CoP displacements during perturbed one-legged stance was used as the most important outcome for reactive balance.

### Quality assessment and statistical analyses

The quality of all eligible studies was assessed using the Appraisal tool for Cross-Sectional Studies (Downes et al., 2016). This tool contains 20 questions that address the study design, the study quality, and the risk of bias. The questions were answered with either “yes,” “no,” or “do not know.” There were seven questions (1, 4, 10, 11, 12, 16, and 18) related to the quality of reporting, 7 questions (2, 3, 5, 8, 17, 19, and 20) related to study design quality, and 6 questions (6, 7, 9, 13, 14, and 15) related to the possible introduction of biases in the study. Three questions (7, 13, and 14) asking for information on non-responders were not included in our analysis because the criterion was not applicable to the studies included in our review. Two independent reviewers (SS, TM) performed the quality assessments of the included studies. When any disagreement between the judges occurred, an additional rating was obtained from a third assessor (RK) to achieve a consensus.

Associations between types of balance were assessed using the Pearson product-moment correlation coefficient (*r*-value). *r*-values derived from different studies were pooled using “Fisher's z' transformation.” In this regard, correlation coefficients were converted to the normally distributed variable *z'* (i.e., z-transformed *r*_*z*_-value). The formula for this transformation is: *z'* = 0.5[ln(1+*r*) - ln(1-*r*)] where ln is the natural logarithm (Kenny, 1987). In addition, the included studies were weighted according to the magnitude of the respective standard error (*SE*). The formula for the calculation of the *SE* is: *SE* = $SE=1/\sqrt{(}N-3)$ where *N* means the respective sample size (Kenny, 1987). Thereafter, we calculated the weighted mean *r*_*z*_-values using Review Manager 5.3 software. For the classification and interpretation of correlation sizes, *r*_*z*_-values were back-transformed to *r*-values. In accordance with the recommendation of Vincent (1995), values of 0 ≤ *r* ≤ 0.69 indicate small, 0.70 ≤ *r* ≤ 0.89 indicate medium, and *r* ≥ 0.90 indicate large sizes of correlation. Lastly, we calculated the differences between the mean back-transformed *r*-values by age groups (Kenny, 1987; Preacher, 2002) using the following formula: $z=(z1-z2)/\sqrt{(1}/(n1-3)+\;1/(n2-3))$. Heterogeneity between studies was assessed using I^2^ and Chi^2^ statistics. Based on the recommendations of Deeks et al. (2008), values of 0% ≤ I^2^ ≤ 40% indicate trivial, 30% ≤ I^2^ ≤ 60% indicate moderate, 50% ≤ I^2^ ≤ 90% indicate substantial, and 75% ≤ I^2^ ≤ 100% shows considerable heterogeneity.

## Results

### Study characteristics

**Figure 1:**
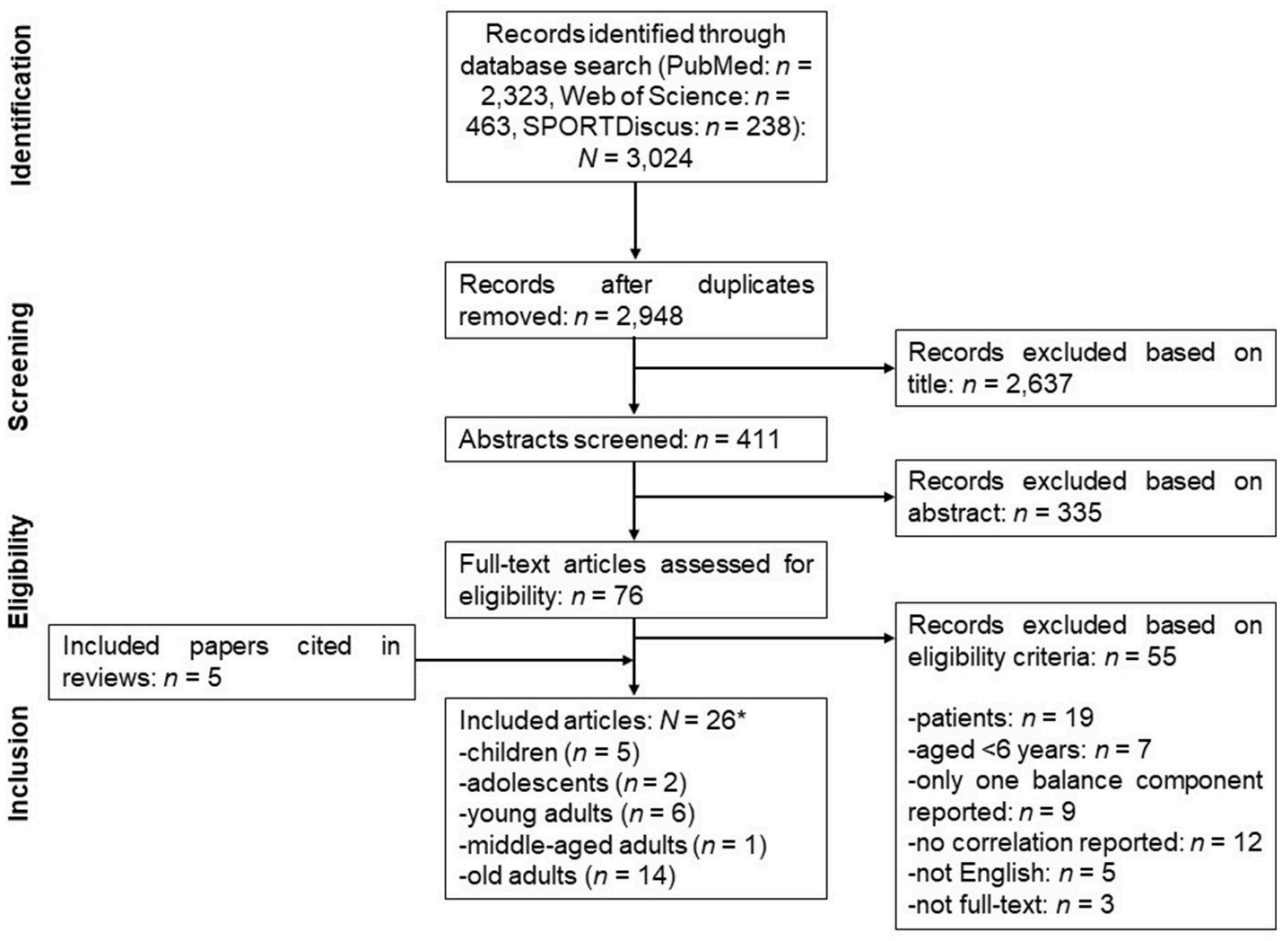
Flow chart describing the systematic literature search.*Two studies investigated multiple age cohorts.

Figure 1 displays a flow chart that illustrates the different stages of the systematic literature search and the selection of studies over the course of the search. The initial search identified 3,024 articles that were potentially eligible for inclusion. After removal of duplicates and exclusion of ineligible articles, 21 articles remained. We identified another 5 articles from the reference lists of already published review articles. Thus, 26 articles were included in the final analysis, whereas 2 of them (Shimada et al., 2003; Granacher et al., 2011a) investigated multiple age cohorts. Table 1 illustrates the main characteristics of the included studies. Of the 26 articles, 4 studies investigated associations between types of balance in children (*n* = 7,016 subjects), 3 studies assessed adolescents (*n* = 383 subjects), 6 studies tested young adults (*n* = 146 subjects), 1 study used middle-aged adults (*n* = 32 subjects), and 14 studies examined old adults (*n* = 1,756 subjects). Irrespective of the age category, 4 studies reported correlations between dynamic steady-state and proactive balance, 2 studies between dynamic steady-state and reactive balance, 15 studies between dynamic and static steady-state balance, 2 studies between proactive and reactive balance, 9 studies between proactive and static steady-state balance, and 11 studies between reactive and static steady-state balance.

**Table 1:** Studies examining associations between types of balance by age group.

**Reference**	**No. of subjects; sex; age, years (range or mean ± SD)**	**Balance type, test, parameter**	**z-transformed *r_z_*-values, explained variance (*r*^2^)**
**CHILDREN (*****N*** = **4)**			
Drowatzky and Zuccato (1967)	50; F; 11–13	sSSB: sideward leap; bass stepping stone test; balance beam test dSSB: two-legged stork stance; diver's stand; stick test	sSSB-dSSB: 0.19, 4%
Granacher and Gollhofer, 2012	30; F (16), M (14); 6–7	sSSB: 20-s two-legged stance with eyes opened on a firmly fixed balance platform, CoP displacement length in ap-/ml-direction dSSB: 20-s two-legged stance with eyes opened on a free moving balance platform, CoP displacement length in ap-/ml-direction	sSSB-dSSB: 0.29, 8%
Humphriss et al., 2011	6,915; F (3,499), M (3,416); 10	sSSB: heel-to-toe stance on a beam, right/left foot forward, eyes open/closed, time; one-legged stance (right/left leg) with eyes open/closed, time dSSB: heel-to-toe beam walking, time	sSSB-dSSB: 0.03, 0%
Muehlbauer et al., 2013a	21; F (8), M (13); 7–10	sSSB: 30-s two-legged stance with eyes opened, CoP displacement length in ap-/ml-direction dSSB: 10-m walk, speed PB: TUG, time; FRT, distance RB: 10-s two-legged stance after perturbation with eyes opened, SO length in ap-/ml-direction	sSSB-PB: 0.41, 17% sSSB-RB: 0.31, 10% dSSB-PB: 0.26, 7% dSSB-RB: 0.22, 5%
**ADOLESCENTS (*****N*** = **3)**			
Granacher and Gollhofer, 2011	28; F (15), M (13); 16–17	sSSB: 30-s one-legged stance with eyes opened, CoP displacement length in ap-/ml-direction RB: 10-s one-legged stance after perturbation with eyes opened, SO length in ap-/ml-direction	sSSB-RB: 0.13, 2%
Ibrahim et al., 2011	330; F (165), M (165); 12–15	sSSB: 60-s one-legged stance with eyes opened/closed, time PB: 10-s jumps with feet together sideways, back and forth over a line	sSSB-PB: 0.33, 11%
Witkowski et al., 2014	25; M; 14–15	sSSB: Flamingo test, time dSSB: Marching test, points	sSSB-dSSB: 0.30, 9%
**YOUNG ADULTS (*****N*** = **6)**			
Granacher et al., 2011a	18; NR; 23 ± 3	sSSB: 30-s two-legged stance with eyes opened, total CoP displacement length dSSB: 10-m walk, coefficient of variation in stride velocity	sSSB-dSSB: 0.05, 0%
Hrysomallis et al., 2006	37; M; 23 ± 4	sSSB: 20-s one-legged stance with eyes opened, CoP displacement length in ml-direction PB: stepping balance task on an unstable surface, CoP displacement length in ml-direction	sSSB-PB: 0.35, 12%
Muehlbauer et al., 2013b	27; F (19), M (8); 23 ± 4	sSSB: 30-s one-legged stance with eyes opened, CoP displacement length in ap-/ml-direction RB: 10-s one-legged stance after perturbation with eyes opened, CoP displacement length in ap-/ml-direction	sSSB-RB: 0.20, 4%
Ringhof and Stein, 2018	24; F; 24 ± 1	sSSB: 30-s one-legged stance with eyes opened, total CoP displacement length PB: one-legged forward jump, time to stabilization RB: one-legged stance after perturbation with eyes opened, time to stabilization	sSSB-PB: 0.15, 2% sSSB-RB: 0.16, 3% PB-RB: 0.16, 3%
Sell, 2012	20; F (10), M (10); 23 ± 3	sSSB: 10-s one-legged stance with eyes opened/closed, SD of ground reaction force in ap-/ml-direction PB: forward/sideward hurdle jump, dynamic postural stability index	sSSB-PB: 0.13, 2%
Shimada et al., 2003	20; NR; 20–32	sSSB: SOT, score RB: perturbed walking on a treadmill, maximum anterior/posterior acceleration of the trunk	sSSB-RB: 0.27, 7%
**MIDDLE-AGED ADULTS (*****N*** = **1)**			
Muehlbauer et al., 2012b	32; F (9), M (23); 56 ± 4	sSSB: 30-s one-legged stance with eyes opened, CoP displacement length in ap-/ml-direction RB: 10-s one-legged stance after perturbation with eyes opened, CoP displacement length in ap-/ml-direction	sSSB-RB: 0.24, 6%
**OLD ADULTS (*****N*** = **14)**			
Callisaya et al., 2009	278; F (124), M (154); 60–86	sSSB: 30-s two-legged stance with eyes closed/opened, total CoP displacement length dSSB: 4.6-m walk, speed, cadence, step length/width	sSSB-dSSB: 0.18, 3%
Callisaya et al., 2010	410; NR; 72 ± 7	sSSB: two-legged stance with eyes closed/opened, postural sway dSSB: 4.6-m walk, speed, step length/width	sSSB-dSSB: 0.23, 5%
Carter et al., 2002	97; F; 69 ± 3	sSSB: SOT, equilibrium score dSSB: figure-eight walk, time	sSSB-dSSB: 0.68, 46%
Forte et al., 2014	57; F (33), M (24); 65–75	sSSB: 30-s Romberg test, total CoP displacement length, velocity; 30-s tandem stance test with eyes opened, total CoP displacement length, velocity dSSB: 10-m walk, gait speed	sSSB-dSSB: 0.03, 0%
Granacher et al., 2011a	18; NR; 74 ± 6	sSSB: 30-s two-legged stance with eyes opened, total CoP displacement length dSSB: 10-m walk, coefficient of variation in stride velocity	sSSB-dSSB: 0.63, 40%
Mackey and Robinovitch, 2005	25; F; 78 ± 7	sSSB: 15-s two-legged stance with eyes opened/closed, total CoP displacement length in ap-/ml-direction RB: balance recovery using a tether release protocol, maximum release angle	sSSB-RB: 0.29, 8%
Mayson et al., 2008	138; NR; 75 ± 7	sSSB: one-legged stance with eyes opened, time dSSB: DGI, score	sSSB-dSSB: 0.19, 4%
Melzer et al., 2009	43, F (27), M (16);78 ± 6	sSSB: 30-s two-legged stance with eyes opened, total CoP displacement area, length, velocity PB: LOS test, total CoP displacement length	sSSB-PB: 0.06, 0%
Miyazaki et al., 2013	124; M; 73 ± 7	sSSB: one-legged stance with eyes opened, time dSSB: 10-m walk, gait speed PB: TUG, time	sSSB-dSSB: 0.42, 18% sSSB-PB: 0.44, 19% dSSB-PB: 0.66, 44%
Muehlbauer et al., 2012a	24; F (13), M (11); 70 ± 5	sSSB: 30-s two-legged stance with eyes opened, CoP displacement length in ap-/ml-direction dSSB: 10-m walk, gait speed PB: TUG, time; FRT, distance RB: 10-s two-legged stance after perturbation with eyes opened, SO length in ap-/ml-direction	sSSB-dSSB: 0.28, 8% sSSB-PB: 0.17, 3% sSSB-RB: 0.04, 0% dSSB-PB: 0.10, 1% dSSB-RB: 0.03, 0%
Owings et al., 2000	79; F (50), M (29); 72 ± 5	sSSB: 20-s two-legged stance with eyes opened, CoP displacement length, speed in ap-/ml-direction PB: LOS test, total CoP displacement length RB: release from forward leaning, maximum recoverable angle; accelerated support surface; mechanically-induced trips	sSSB-PB: 0.18, 3% sSSB-RB: 0.08, 1% PB-RB: 0.14, 2%
Ringsberg et al., 1999	230; F; 75	sSSB: one-legged stance, time; 20-s two-legged stance with eyes opened/closed, total CoP displacement length dSSB: 30-m walk, time, cadence RB: 20-s two-legged stance with eyes opened on a moving platform, total CoP displacement length	sSSB-dSSB: 0.55, 30% sSSB-RB: 0.23, 5%
Shimada et al., 2003	20; NR; 65–79	sSSB: SOT, score RB: perturbed walking on a treadmill, maximum anterior/posterior acceleration of the trunk	sSSB-RB: 0.30, 9%
Shimada et al., 2011	213; F (130), M (83); 65–96	sSSB: 120-s one-legged stance with eyes opened, time dSSB: 6-m walk, time PB: TUG, time	sSSB-dSSB: 0.18, 3% sSSB-PB: 0.41, 17% dSSB-PB: 0.85, 72%

*ap, anterior-posterior; CoP, center of pressure; DGI, dynamic gait index; dSSB, dynamic steady-state balance; F, female; FRT, Functional-Reach-Test; LOS, Limits-of-stability-Test; M, male; ml, medio-lateral; NR, not reported; PB, proactive balance; RB, reactive balance; sSSB, static steady-state balance; SD, standard deviation; SO, summed oscillations; SOT, sensory organization test; TUG, Timed-Up-and-Go-Test*.

### Quality of the included studies

Quality assessment revealed that the majority of studies included in our review met the criteria for (a) study design, (b) study quality, and (c) risk of bias above average. More specifically, twenty-five of the 26 included studies fulfilled ≥4 out of 7 criteria evaluating quality of study reports (Table S1, online supplement). Concerning quality of study design, ≥4 out of 7 criteria were fulfilled by 24 studies. Lastly, 20 studies fulfilled ≥2 out of 3 criteria with respect to risk of bias.

### Correlations between dynamic steady-state and proactive balance

**Figure 2:**
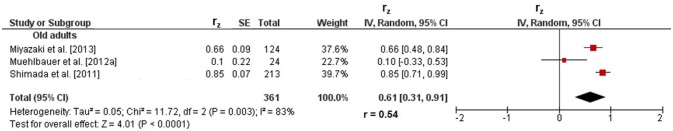
Pearson's *r*-values (z-transformed) for correlations between dynamic steady-state and proactive balance in old adults. *CI* confidence interval, *df* degrees of freedom, *r* back-transformed Pearson's correlation coefficients, *r*_*z*_ weighted z-transformed Pearson's correlation coefficients, *SE* standard error, *IV* inverse variance.

Figure 2 illustrates the correlations of dynamic steady-state with proactive balance in old adults. The weighted mean *r*_z_-value amounted to 0.61 and was accompanied with considerable heterogeneity (I^2^ = 83%, Chi^2^ = 11.72, *df* = 2, *p* = 0.003). The back-transformed *r*-value of 0.54 indicated a small-sized correlation. Only one study (Muehlbauer et al., 2013a) reported a small correlation (*r*_z_ = 0.26, *r* = 0.25) between dynamic steady-state and proactive balance in children (Table 1). No study reported associations of dynamic steady-state with proactive balance in adolescents, young, and middle-aged adults.

### Correlations between dynamic steady-state and reactive balance

In children (*r*_z_ = 0.22, *r* = 0.22) (Muehlbauer et al., 2013a) and in old adults (*r*_z_ = 0.03, *r* = 0.03) (Muehlbauer et al., 2012a), only one study reported small-sized correlations between dynamic steady-state and reactive balance (Table 1). No study reported associations of dynamic steady-state with reactive balance in adolescents, young and middle-aged adults.

### Correlations between dynamic and static steady-state balance

**Figure 3:**
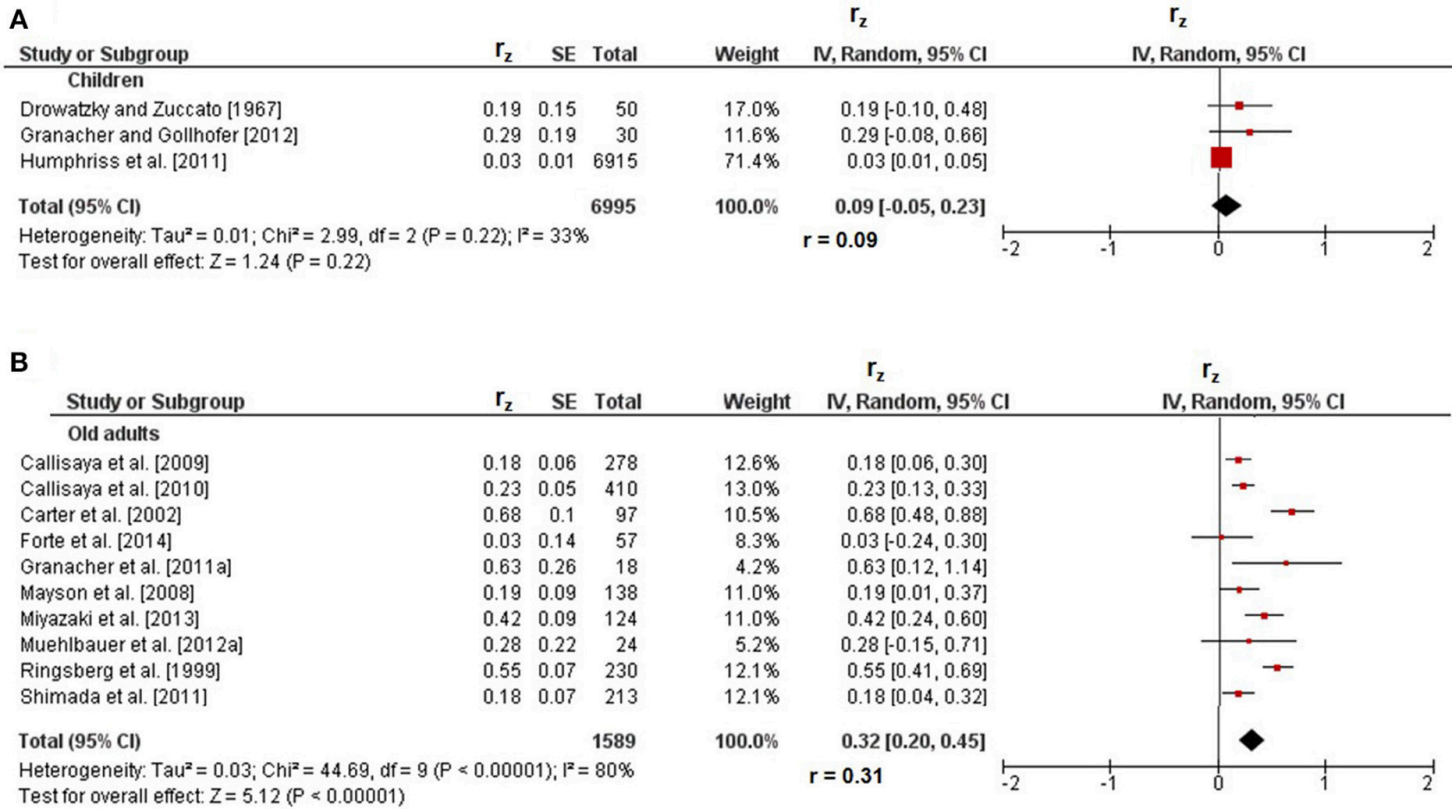
Pearson's *r*-values (z-transformed) for correlations between dynamic and static steady-state balance in children **(A)** and old adults **(B)**. *CI*, confidence interval; *df*, degrees of freedom; *r*, back-transformed Pearson's correlation coefficients; *r*_*z*_, weighted z-transformed Pearson's correlation coefficients; *SE*, standard error; *IV*, inverse variance.

Figure 3 displays the correlations of dynamic with static steady-state balance. Weighted mean *r*_z_-values amounted to 0.09 in children (I^2^ = 33%, Chi^2^ = 2.99, *df* = 2, *p* = 0.22) and to 0.32 in old adults (I^2^ = 80%, Chi^2^ = 44.69, *df* = 9, *p* < 0.001) and were accompanied with moderate to considerable heterogeneity. Back-transformed *r*-values of 0.09 and 0.31 indicated small-sized correlations, respectively. In adolescents (*r*_z_ = 0.30, *r* = 0.29) (Witkowski et al., 2014) and in young adults (*r*_z_ = 0.05, *r* = 0.05) (Granacher et al., 2011a), only one study reported small-sized correlations between dynamic and static steady-state balance (Table 1). No study reported associations of dynamic with static steady-state balance in middle-aged adults.

### Correlations between proactive and reactive balance

In young (*r*_z_ = 0.16, *r* = 0.16) (Ringhof and Stein, 2018) and in old adults (*r*_z_ = 0.14, *r* = 0.14) (Owings et al., 2000), only one study reported small-sized correlations between proactive and reactive balance (Table 1). No study reported associations of proactive with reactive balance in children, adolescents, and middle-aged adults.

### Correlations between proactive and static steady-state balance

**Figure 4:**
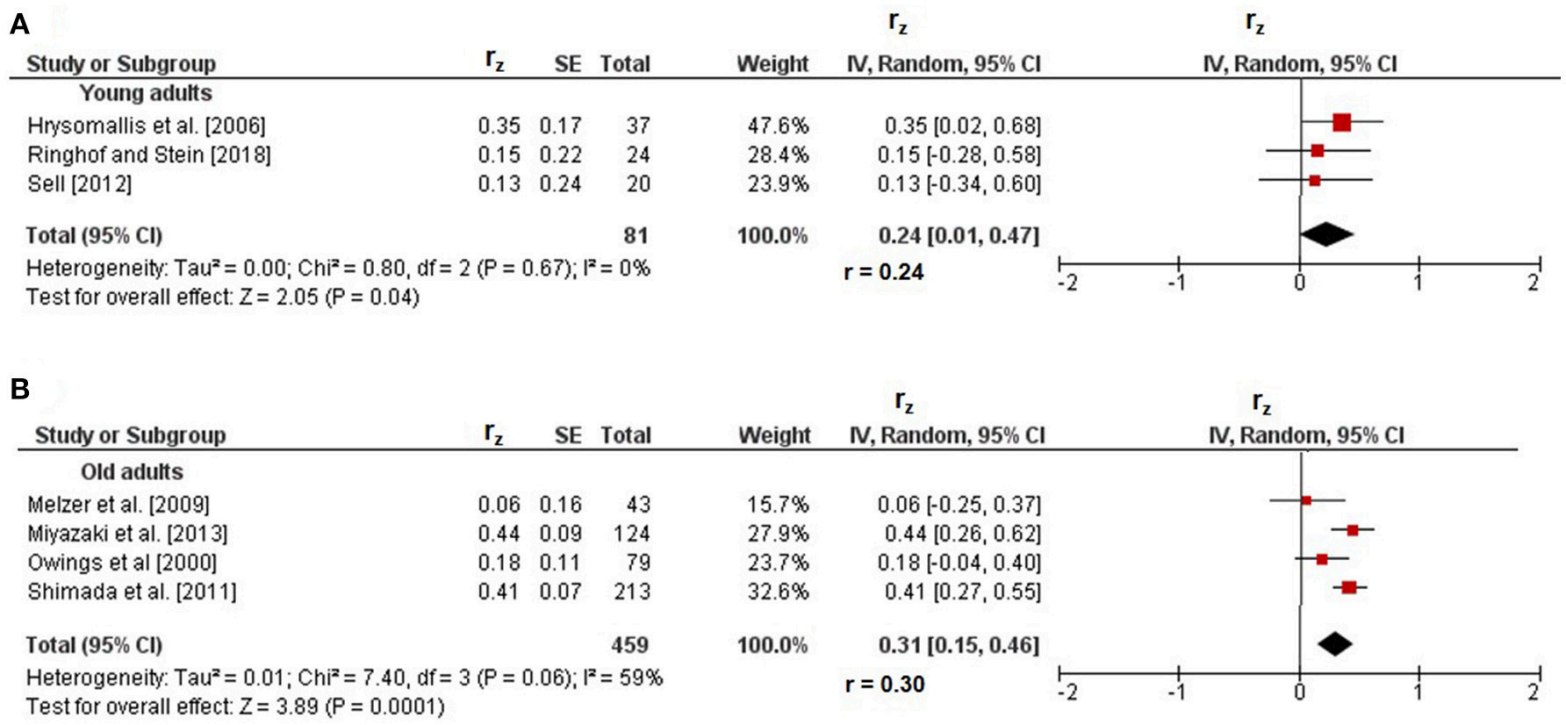
Pearson's *r*-values (z-transformed) for correlations between proactive and static steady-state balance in young **(A)** and old adults **(B)**. *CI*, confidence interval; *df*, degrees of freedom; *r*, back-transformed Pearson's correlation coefficients; *r*_*z*_, weighted z-transformed Pearson's correlation coefficients; *SE*, standard error; *IV*, inverse variance.

Figure 4 illustrates the correlations of proactive with static steady-state balance. Weighted mean *r*_z_-values amounted to 0.24 in young adults (I^2^ = 0%, Chi^2^ = 0.80, *df* = 2, *p* = 0.67) and to 0.31 in old adults (I^2^ = 59%, Chi^2^ = 7.40, *df* = 3, *p* = 0.06) and were accompanied with trivial to substantial heterogeneity. The respective back-transformed *r*-values of 0.24 and 0.30 indicated small-sized correlations. In children (*r*_z_ = 0.41, *r* = 0.39) (Muehlbauer et al., 2013a) and in adolescents (*r*_z_ = 0.33, *r* = 0.32) (Ibrahim et al., 2013), only one study reported small-sized correlations between proactive and static steady-state balance (Table 1). No study reported associations of proactive with static steady-state balance in middle-aged adults.

### Correlations between reactive and static steady-state balance

**Figure 5:**
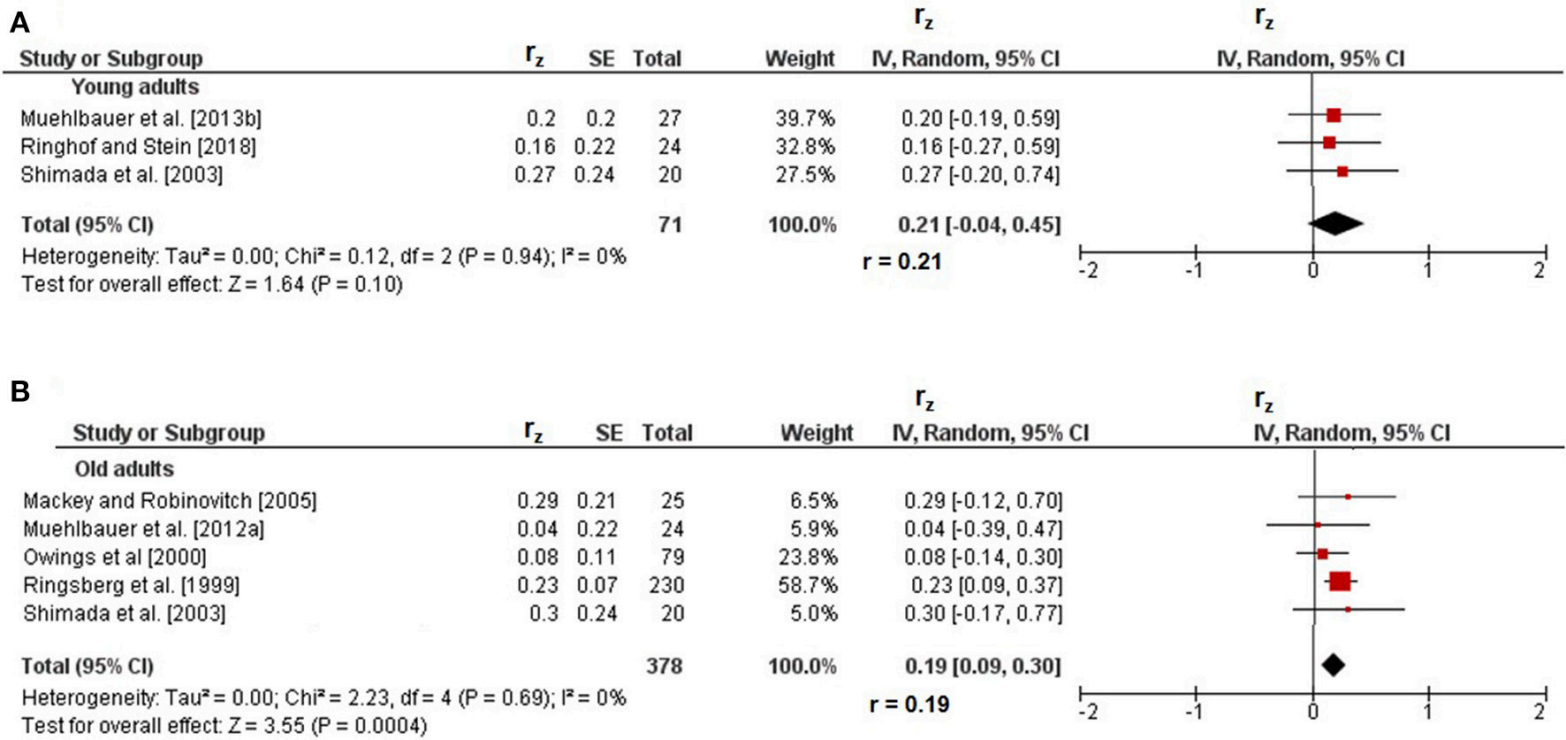
Pearson's *r*-values (z-transformed) for correlations between reactive and static steady-state balance in young **(A)** and old adults **(B)**. *CI*, confidence interval; *df*, degrees of freedom; *r*, back-transformed Pearson's correlation coefficients; *r*_*z*_, weighted z-transformed Pearson's correlation coefficients; *SE*, standard error; *IV*, inverse variance.

Figure 5 displays the correlations of reactive with static steady-state balance. Weighted mean *r*_z_-values amounted to 0.21 in young adults (I^2^ = 0%, Chi^2^ = 0.12, *df* = 2, *p* = 0.94) and to 0.19 in old adults (I^2^ = 0%, Chi^2^ = 2.23, *df* = 4, *p* = 0.69) and were accompanied with trivial heterogeneity. Back-transformed *r*-values of 0.21 and 0.19 indicated small-sized correlations. In children (*r*_z_ = 0.31, *r* = 0.30) (Muehlbauer et al., 2013a), adolescents (*r*_z_ = 0.13, *r* = 0.13) (Granacher and Gollhofer, 2011), and middle-aged adults (*r*_z_ = 0.24, *r* = 0.24) (Muehlbauer et al., 2012b), only one study reported small-sized correlations between reactive and static steady-state balance (Table 1).

### Age comparison of correlations between types of balance performance

Statistically significant differences between age groups were obtained for the association of dynamic with static steady-state balance only. More precisely, the *r*-value in children (*r* = 0.09) was significantly smaller (*z* = 8.28, *p* < 0.001) than that in old adults (*r* = 0.31). Additional age comparisons of static steady-state balance with proactive (*z* = 0.53, *p* = 0.60) and reactive (*z* = 0.16, *p* = 0.87) balance did not reveal significant differences in young compared to old adults.

## Discussion

This systematic review and meta-analysis quantified and statistically compared associations between types of balance in healthy individuals across the lifespan. The main findings can be summarized as follows. First, we found exclusively small-sized correlations between types of balance in children, adolescents, young, middle-aged, and old adults. This finding was independent from the investigated type of balance (i.e., dynamic/static steady-state, proactive, and reactive balance). Second, we detected significantly smaller correlations between dynamic and static steady-state balance in children compared to old adults. However, the analyses failed to detect further significant age differences for associations between other types of balance.

### Associations between types of balance performance in healthy individuals across the lifespan

Our finding of exclusively small-sized correlations between types of balance contradicts the notion of balance as a “general ability,” as indicated in textbooks (Fleishman, 1964; Schnabel et al., 2014; Meinel and Schnabel, 2018), and is in accordance with the presumption of Shumway-Cook and Woollacott (2016) who identified various types of balance performance (i.e., dynamic/static steady-state, proactive, and reactive balance). Based on the observation of small-sized correlations, it is suggested that types of balance performance are relatively independent and task-specific and thus, should be considered individually. For example, if a person shows a high amount of dynamic steady-state balance (e.g., fast gait speed in the 10-m walk test), an experimenter would not be able to predict how well that person would perform on a test of proactive balance (e.g., distance in the Functional-Reach-Test). Thus, if the goal is to assess balance performance, practitioners are not advised to only use one test, but rather utilize test batteries assessing different types of balance. Concerning the implication for training, the finding of low correlations indicates that programs including exercises for dynamic/static steady-state, proactive, and reactive types of balance should be applied if the goal is to enhance balance performance.

A possible reason for the observed small-sized correlations between types of balance could be differences in the specific task requirements. That is, during static steady-state balance tasks, such as one-legged standing, the base of support (i.e., foot) and the ground remain stationary as only the center of mass moves. However, during walking, as a representative of a dynamic steady-state balance task, the base of support and the center of mass shift, which provides different requirements to the involved neurophysiological structures than quiet standing. In this regard, Lau et al. (2014) investigated electrocortical activity using high-density electroencephalography during standing and walking on a treadmill in healthy young adults (age range: 20–31 years). They found that connections involving the sensorimotor cortex were significantly weaker during walking compared to standing. The authors interpreted this finding as a greater cortical involvement during standing than walking, because spinal neural networks play a larger role in the control of locomotion than stance. Further, the tested individuals might differentially experience balance task intensity and difficulty. For instance, normal walking or one-legged standing could be a low intensity balance task condition for young and middle-aged adults, but a high intensity task condition for children, adolescents, and/or old adults. Moreover, different mechanisms are involved for the control of proactive (e.g., distance in the Functional-Reach-Test) and reactive (e.g., postural sway during perturbed unipedal stance) balance (Riemann and Lephart, 2002). In the first case, feedforward control is necessary that involves the anticipation of a predicted postural disturbance during maximal forward leaning and the initiation of adequate muscle responses to prevent loss of balance. On the contrary, feedback control is characterized by the initiation of sufficient muscle responses after balance loss to compensate an unpredicted postural disturbance during one-legged standing and to avoid falling. In this respect, recent studies (Wälchli et al., 2017; Fujio et al., 2018) showed that the central nervous system differently prepares postural responses in expected compared to unexpected stance perturbations. For instance, Fujio et al. (2018) examined motor-evoked potential (MEP) induced by transcranial magnetic stimulation during expected (via acoustic signal) and unexpected (no signal) perturbations, while standing on a moveable platform in healthy young adults (mean age: 27 ± 2 years). As a result, the MEP for the tibialis anterior muscle was significantly enhanced under expected compared to the unexpected stance perturbation. Fujio and colleagues concluded that a prediction of an upcoming perturbation of standing balance modulates the excitability of corticospinal pathways. Additional physiological (i.e., fatigue) and psychological (i.e., attention, motivation) factors (Zech et al., 2012; Muehlbauer et al., 2013a) that are known to affect postural control might also have contributed to a larger or lesser amount while testing one compared to another type of balance and thus resulting in small-sized correlations. In summary, the observed small correlations between types of balance across lifespan are likely to reflect (i) differences in balance task complexity, difficulty, and/or intensity, (ii) discrepancies in the neurophysiological mechanisms involved in postural control, and (iii) the influence of additional physiological and psychological factors. Thus, the notion of balance as a “general ability” cannot be completely ruled out and further research is needed to examine whether these aspects masked the obtained correlations.

### Age comparison of correlations between types of balance performance

Significant age differences were found for associations between dynamic and static steady-state balance in children compared to old adults. More specifically, the correlation coefficient was smaller in children compared to old adults. Based on this finding, one may argue that maturation/age have an effect on the association of selected types of balance. However, we could not detect further significant age differences in the relationship between other types of balance. Moreover, a closer look on the studies involved in the comparison that revealed significant age differences shows that fairly low correlation coefficients were reported in the study of Humphriss et al. (2011). For example, the association between dynamic (i.e., time for heel-to-toe beam walking) and static (i.e., time for the one-legged stance with eyes opened/closed) steady-state balance resulted in *r*-values of −0.0163 and −0.0531, respectively. When excluding the study by Humphriss et al. (2011) from our analysis, results indicated an increase of the back-transformed mean *r*-value from 0.09 to 0.23 and the formerly significant difference in associations between dynamic and static steady-state balance in children compared to old adults did not reach significance (*z* = 0.74, *p* = 0.46). Thus, methodological inconsistencies (i.e., no study directly compared several age cohorts using identical balance tests, parameters) between the involved studies could have also caused the significant age differences. As a consequence, further research is needed to investigate whether associations between types of balance are affected by maturation/age or methodological inconsistencies. To investigate whether the detected age differences between dynamic and static steady-state balance in children compared to old adults truly exist, it is recommended to conduct a series of single studies quantifying and statistically comparing correlations between various types of balance in children, adolescents, young, middle-aged, and old adults using identical tests and parameters.

## Conclusions

The present systematic review and meta-analysis revealed exclusively small-sized correlations between types of balance performance in children, adolescents, young, middle-aged, and old adults. Findings indicate that balance performance seems to be task-specific rather than a “general ability.” Thus, we advise practitioners to apply a test battery and not a single test for balance assessment. Further, multiple exercises including dynamic/static steady-state, proactive, and reactive types of balance should be used during balance training to target each balance dimension individually. In addition, we found significantly smaller correlation coefficients for the association of dynamic with static steady-state balance in children compared to old adults. This implies that maturation/age may have an effect on the association between selected types of balance. Yet, methodological inconsistencies (i.e., indirect age comparisons) between the involved studies could have also caused the significant age differences and thus further research is needed to investigate whether the observed age differences could be replicated.

## Author contributions

RK worked on study design and manuscript preparation. SS assisted on data collection, data analysis, and worked on manuscript preparation. TM worked on study design, data collection, data analysis, and manuscript preparation.

### Conflict of interest statement

The authors declare that the research was conducted in the absence of any commercial or financial relationships that could be construed as a potential conflict of interest.

## References

[B1] Bohannon R. W. (2006). Reference values for the timed up and go test: a descriptive meta-analysis. J. Geriatr. Phys. Ther. 29, 64–68. 10.1519/00139143-200608000-00004 16914068

[B2] Bouche P. Cattelin F. Saint-Jean O. Léger J. M. Queslati S. Guez D.. (1993). Clinical and electrophysiological study of the peripheral nervous system in the elderly. J. Neurol. 240, 263–268. 10.1007/BF00838158 8326328

[B3] Callisaya M. L. Blizzard L. McGinley J. L. Schmidt M. D. Srikanth V. K. (2010). Sensorimotor factors affecting gait variability in older people–a population-based study. J. Gerontol. A Biol. Sci. Med. Sci. 65, 386–392. 10.1093/gerona/glp184 19939910

[B4] Callisaya M. L. Blizzard L. Schmidt M. D. McGinley J. L. Lord S. R. Srikanth V. K. (2009). A population-based study of sensorimotor factors affecting gait in older people. Age Ageing 38, 290–295. 10.1093/ageing/afp017 19264860

[B5] Carter N. D. Khan K. M. Mallinson A. Janssen P. A. Heinonen A. Petit M. A.. (2002). Knee extension strength is a significant determinant of static and dynamic balance as well as quality of life in older community-dwelling women with osteoporosis. Gerontology 48, 360–368. 10.1159/000065504 12393951

[B6] Deeks J. J. Higgins J. P. T. Altman D. G. (2008). Chapter 9: Analysing data and undertaking meta-analyses, in Cochrane Handbook for Systematic Reviews of Interventions, eds Higgins J. P. T. Green S. (Chichester, UK: The Cochrane Collaboration), 1–43.

[B7] Downes M. J. Brennan M. L. Williams H. C. Dean R. S. (2016). Development of a critical appraisal tool to assess the quality of cross-sectional studies (AXIS). BMJ Open 6:e011458. 10.1136/bmjopen-2016-011458 27932337 PMC5168618

[B8] Drowatzky J. N. Zuccato F. C. (1967). Interrelationships between selected measures of static and dynamic balance. Res. Q. 38, 509–510. 5235513

[B9] Fleishman E. A. (1964). The Structure and Measurement of Physical Fitness. Englewood Cliffs, NJ: Prentice-Hall.

[B10] Forte R. Boreham C. A. De Vito G. Ditroilo M. Pesce C. (2014). Measures of static postural control moderate the association of strength and power with functional dynamic balance. Aging Clin. Exp. Res. 26, 645–653. 10.1007/s40520-014-0216-0 24715650

[B11] Fousekis K. Tsepis E. Poulmedis P. Athanasopoulos S. Vagenas G. (2011). Intrinsic risk factors of non-contact quadriceps and hamstring strains in soccer: a prospective study of 100 professional players. Br. J. Sports Med. 45, 709–714. 10.1136/bjsm.2010.077560 21119022

[B12] Fujio K. Obata H. Kitamura T. Kawashima N. Nakazawa K. (2018). Corticospinal excitability is modulated as a function of postural perturbation predictability. Front. Hum. Neurosci. 12:68. 10.3389/fnhum.2018.00068 29535618 PMC5835041

[B13] Granacher U. Bridenbaugh S. A. Muehlbauer T. Wehrle A. Kressig R. W. (2011a). Age-related effects on postural control under multi-task conditions. Gerontology 57, 247–255. 10.1159/000322196 20980734

[B14] Granacher U. Gollhofer A. (2011). Is there an association between variables of postural control and strength in adolescents? J. Strength Cond. Res. 25, 1718–1725. 10.1519/JSC.0b013e3181dbdb08 21358425

[B15] Granacher U. Gollhofer A. (2012). Is there an association between variables of postural control and strength in prepubertal children? J. Strength Cond. Res. 26, 210–216. 10.1519/JSC.0b013e31821b7c30 22201695

[B16] Granacher U. Muehlbauer T. Gollhofer A. Kressig R. W. Zahner L. (2011b). An intergenerational approach in the promotion of balance and strength for fall prevention - a mini-review. Gerontology 57, 304–315. 10.1159/000320250 20720401

[B17] Hirabayashi S. Iwasaki Y. (1995). Developmental perspective of sensory organization on postural control. Brain Dev. 17, 111–113. 10.1016/0387-7604(95)00009-Z 7542846

[B18] Hrysomallis C. (2007). Relationship between balance ability, training and sports injury risk. Sports Med. 37, 547–556. 10.2165/00007256-200737060-00007 17503879

[B19] Hrysomallis C. McLaughlin P. Goodman C. (2006). Relationship between static and dynamic balance tests among elite Australian Footballers. J. Sci. Med. Sport 9, 288–291. 10.1016/j.jsams.2006.05.021 16844414

[B20] Humphriss R. Hall A. May M. Macleod J. (2011). Balance ability of 7 and 10 year old children in the population: results from a large UK birth cohort study. Int. J. Pediatr. Otorhinolaryngol. 75, 106–113. 10.1016/j.ijporl.2010.10.019 21074865

[B21] Ibrahim A. I. Muaidi Q. I. Abdelsalam M. S. Hawamdeh Z. M. Alhusaini A. A. (2013). Association of postural balance and isometric muscle strength in early- and middle-school-age boys. J. Manipulative Physiol. Ther. 36, 633–643. 10.1016/j.jmpt.2013.08.009 24144424

[B22] Ibrahim H. Heard N. P. Blanksby B. (2011). Exploring the general motor ability construct. Percept. Mot. Skills 113, 491–508. 10.2466/03.06.19.25.PMS.113.5.491-508 22185064

[B23] Kenny D. A. (1987). Statistics for the Social and Behavioral Sciences. London: Longman.

[B24] Lau T. M. Gwin J. T. Ferris D. P. (2014). Walking reduces sensorimotor network connectivity compared to standing. J. Neuroeng. Rehabil. 11:14. 10.1186/1743-0003-11-14 24524394 PMC3929753

[B25] Mackey D. C. Robinovitch S. N. (2005). Postural steadiness during quiet stance does not associate with ability to recover balance in older women. Clin. Biomech. 20, 776–783. 10.1016/j.clinbiomech.2005.05.002 16006022

[B26] Maisonobe T. Hauw J. J. Dani S. U. Hori A. Walter G. F. (1997). Changes in the peripheral nervous system, in Principles of Neural Aging, eds Dani S. U. Hori A. Walter G. G. (Amsterdam: Elsevier), 304–316.

[B27] Mayson D. J. Kiely D. K. LaRose S. I. Bean J. F. (2008). Leg strength or velocity of movement: which is more influential on the balance of mobility limited elders? Am. J. Phys. Med. Rehabil. 87, 969–976. 10.1097/PHM.0b013e31818dfee5 19033758 PMC2731581

[B28] Meinel K. Schnabel G. (2018). Bewegungslehre Sportmotorik: Abriss einer Theorie der sportlichen Motorik unter pädagogischem Aspekt. Aachen: Meyer and Meyer.

[B29] Melzer I. Benjuya N. Kaplanski J. Alexander N. (2009). Association between ankle muscle strength and limit of stability in older adults. Age Ageing 38, 119–123. 10.1093/ageing/afn249 19029102 PMC3104613

[B30] Miyazaki J. Murata S. Horie J. Uematsu A. Hortobágyi T. Suzuki S. (2013). Lumbar lordosis angle (LLA) and leg strength predict walking ability in elderly males. Arch. Gerontol. Geriatr. 56, 141–147. 10.1016/j.archger.2012.09.004 23063093

[B31] Muehlbauer T. Besemer C. Wehrle A. Gollhofer A. Granacher U. (2012a). Relationship between strength, power and balance performance in seniors. Gerontology 58, 504–512. 10.1159/000341614 22922168

[B32] Muehlbauer T. Besemer C. Wehrle A. Gollhofer A. Granacher U. (2013a). Relationship between strength, balance and mobility in children aged 7-10 years. Gait Posture 37, 108–112. 10.1016/j.gaitpost.2012.06.022 22832473

[B33] Muehlbauer T. Gollhofer A. Granacher U. (2012b). Relationship between measures of balance and strength in middle-aged adults. J. Strength Cond. Res. 26, 2401–2407. 10.1519/JSC.0b013e31823f8c41 22076099

[B34] Muehlbauer T. Gollhofer A. Granacher U. (2013b). Association of balance, strength, and power measures in young adults. J. Strength Cond. Res. 27, 582–589. 10.1519/JSC.0b013e31825c2bab 23443216

[B35] Muehlbauer T. Gollhofer A. Granacher U. (2015). Associations between measures of balance and lower-extremity muscle strength/power in healthy individuals across the lifespan: a systematic review and meta-analysis. Sports Med. 45, 1671–1692. 10.1007/s40279-015-0390-z 26412212 PMC4656701

[B36] Owings T. M. Pavol M. J. Foley K. T. Grabiner M. D. (2000). Measures of postural stability are not predictors of recovery from large postural disturbances in healthy older adults. J. Am. Geriatr. Soc. 48, 42–50. 10.1111/j.1532-5415.2000.tb03027.x 10642020

[B37] Peterson M. L. Christou E. Rosengren K. S. (2006). Children achieve adult-like sensory integration during stance at 12-years-old. Gait Posture 23, 455–463. 10.1016/j.gaitpost.2005.05.003 16002294

[B38] Preacher K. J. (2002). Calculation for the Test of the Difference Between Two Independent Correlation Coefficients [Computer software]. Available online at: http://quantpsy.org (Accessed August 19, 2018).

[B39] Razmus I. Wilson D. Smith R. Newman E. (2006). Falls in hospitalized children. Pediatr. Nurs. 32, 568–572. 17256296

[B40] Riemann B. L. Lephart S. M. (2002). The sensorimotor system, part I: the physiologic basis of functional joint stability. J. Athl. Train. 37, 71–79. 16558670 PMC164311

[B41] Ringhof S. Stein T. (2018). Biomechanical assessment of dynamic balance: specificity of different balance tests. Hum. Mov. Sci. 58, 140–147. 10.1016/j.humov.2018.02.004 29438911

[B42] Ringsberg K. Gerdhem P. Johansson J. Obrant K. J. (1999). Is there a relationship between balance, gait performance and muscular strength in 75-year-old women? Age Ageing 28, 289–293. 10.1093/ageing/28.3.289 10475866

[B43] Rubenstein L. Z. (2006). Falls in older people: epidemiology, risk factors and strategies for prevention. Age Ageing 35(Suppl. 2), ii37–ii41. 10.1093/ageing/afl084 16926202

[B44] Schnabel G. Harre H. D. Krug J. (2014). Trainingslehre-Trainingswissenschaft: Leistung-Training-Wettkampf. Aachen: Meyer and Meyer.

[B45] Sell T. C. (2012). An examination, correlation, and comparison of static and dynamic measures of postural stability in healthy, physically active adults. Phys. Ther. Sport 13, 80–86. 10.1016/j.ptsp.2011.06.006 22498148

[B46] Shimada H. Obuchi S. Kamide N. Shiba Y. Okamoto M. Kakurai S. (2003). Relationship with dynamic balance function during standing and walking. Am. J. Phys. Med. Rehabil. 82, 511–516. 10.1097/01.PHM.0000064726.59036.CB 12819538

[B47] Shimada H. Tiedemann A. Lord S. R. Suzukawa M. Makizako H. Kobayashi K.. (2011). Physical factors underlying the association between lower walking performance and falls in older people: a structural equation model. Arch. Gerontol. Geriatr. 53, 131–134. 10.1016/j.archger.2010.11.003 21145119

[B48] Shumway-Cook A. Woollacott M. H. (1985). The growth of stability: postural control from a development perspective. J. Mot. Behav. 17, 131–147. 10.1080/00222895.1985.10735341 15140688

[B49] Shumway-Cook A. Woollacott M. H. (2016). Motor Control: Translating Research Into Clinical Practice. Philadelphia, PA: Lippincott Williams and Wilkins.

[B50] Springer B. A. Marin R. Cyhan T. Roberts H. Gill N. W. (2007). Normative values for the unipedal stance test with eyes open and closed. J. Geriatr. Phys. Ther. 30, 8–15. 10.1519/00139143-200704000-00003 19839175

[B51] Steindl R. Kunz K. Schrott-Fischer A. Scholtz A. W. (2006). Effect of age and sex on maturation of sensory systems and balance control. Dev. Med. Child Neurol. 48, 477–482. 10.1017/S0012162206001022 16700940

[B52] Terao S. Sobue G. Hashizume Y. Li M. Inagaki T. Mitsuma T. (1996). Age-related changes in human spinal ventral horn cells with special reference to the loss of small neurons in the intermediate zone: a quantitative analysis. Acta Neuropathol. 92, 109–114. 10.1007/s004010050497 8841655

[B53] Vincent W. J. (1995). Statistics in Kinesiology. Champaign, IL: Human Kinetics.

[B54] Wälchli M. Tokuno C. D. Ruffieux J. Keller M. Taube W. (2017). Preparatory cortical and spinal settings to counteract anticipated and non-anticipated perturbations. Neuroscience 365, 12–22. 10.1016/j.neuroscience.2017.09.032 28951323

[B55] Wang H. K. Chen C. H. Shiang T. Y. Jan M. H. Lin K. H. (2006). Risk-factor analysis of high school basketball-player ankle injuries: a prospective controlled cohort study evaluating postural sway, ankle strength, and flexibility. Arch. Phys. Med. Rehabil. 87, 821–825. 10.1016/j.apmr.2006.02.024 16731218

[B56] Witkowski K. Maslinski J. Remiarz A. (2014). Static and dynamic balance in 14-15 year old boys training judo and in their non-active peers. Arch. Budo 10, 12–24.

[B57] Woollacott M. H. Shumway-Cook A. (1990). Changes in posture control across the life span–a systems approach. Phys. Ther. 70, 799–807. 10.1093/ptj/70.12.799 2236223

[B58] Woollacott M. H. Shumway-Cook A. (1994). Maturation of Feedback Control of Posture and Equilibrium, in Motor Development in Children, eds Fedrizzi E. Avanzini G. Crenna P. (New Barnet: John Libbey and Company Ltd).

[B59] Zech A. Steib S. Hentschke C. Eckhardt H. Pfeifer K. (2012). Effects of localized and general fatigue on static and dynamic postural control in male team handball athletes. J. Strength Cond. Res. 26, 1162–1168. 10.1519/JSC.0b013e31822dfbbb 22446681

[B60] Zemkova E. (2014). Sport-specific balance. Sports Med. 44, 579–590. 10.1007/s40279-013-0130-1 24293269

